# Notes on an Epidemic of Measles

**Published:** 1891-06

**Authors:** Frederick Stanbro

**Affiliations:** Springville, N. Y.


### NOTES ON AN EPIDEMIC OF MEASLES.

By FREDERICK STANBRO, M. D., Springville, N. Y.

I WRITE these notes, not because Rubeola is such a rare disease, but in order to put on record certain irregularities observed during this epidemic, which, I trust, will be of interest, and may be of service, to the general practitioner.

Beginning in the last week of January, 1891, and continuing seven weeks, there has been a severe epidemic of measles in this region. No such epidemic has been observed here in fifteen years, both as regards severity, numbers attacked, and small mortality.

All unprotected persons have been attacked, and six authentic cases I have observed which have suffered a second attack. These six persons had measles before, under the observation and care of physicians in this vicinity, and there can be no mistake. Three had mild attacks from three to ten years ago, and three had severe attacks seven years ago. Each of these six cases had measles under my observation this season, and all had it in the severest form, the prodromes, rash, catarrhal symptoms, all being typical. I lay special stress on these cases, as there seems to be some doubt on this subject among medical minds, as well as among the laity.

Of the 110 cases observed and treated, I find sixty males and fifty females; of these, twenty-four were adults and six were infants under the age of nine months.

The period of incubation was about nine days, but the eruptive stage was wonderfully delayed, the rash not appearing until a week had passed, in the majority of cases, and in two cases of young ladies, aged respectively sixteen and eighteen years, the rash did not appear until nine and fourteen days had passed, during which time they were confined to the bed with the usual symptoms of the prodromal stage—viz., Malaise, headache, bone-ache, slight fever, etc.

During this period of "invasion" the patients were all ill and suffered from aching bones and muscles, constipation, nausea, headache, and other symptoms similar to those of "la grippe" of last year. A slight rise of temperature would be observed, and for about three days before the appearance of the rash the coryzal symptoms and the peculiar hacking cough would appear.

The temperature was extremely high as a rule, being 103° F. in most of the cases, 104° F. in twenty cases, 105° F. in ten cases, and $105^{50}$ F. in three cases.

The pulse was correspondingly high, running from 120 to 160

in the severest cases. Slight congestion of kidneys occurred in most of the cases during the few days before the appearance of the rash, and delirium was a common symptom.

In a few cases, earache was so severe as to call for local treatment.

*Sequelæ.*—Catarrhal pneumonia attacked one patient weakened by the measles, and whooping cough occurred in nine cases. The severe coughing caused a direct inguinal hernia in a young man about sixteen years of age. Otherwise all the cases recovered, not one of the 110 dying, and only one is now under my observation.

*Treatment.*—The simpler cases demanded no treatment, except light diet and some calming expectorant.

The more severe cases were kept in warm, darkened rooms on strictly milk diet. High fever was relieved or controlled by tr. aconite, quinine and tepid baths. Constipation relieved only by warm enemata. Dysuria, or suppression of urine, by spirits of nitre and other simple diuretics.

The conjunctivitis required nothing but warm lotions, and the general pruritus was relieved by inunctions of vaseline or lard.

Only a few required any tonic treatment during convalescence.

